# Sortilin is associated with the chlamydial inclusion and is modulated during infection

**DOI:** 10.1242/bio.016485

**Published:** 2016-03-09

**Authors:** Wei Xuan Teo, Markus Charles Kerr, Wilhelmina May Huston, Rohan David Teasdale

**Affiliations:** 1 Institute for Molecular Biosciences, The University of Queensland, Brisbane, Queensland 4067, Australia; 2 School of Life Sciences, University of Technology Sydney, Broadway, New South Wales 2007, Australia

**Keywords:** *Chlamydia trachomatis*, Ceramide, Sortilin, Acid sphingomyelinase

## Abstract

*Chlamydia* species are obligate intracellular pathogens that have a major impact on human health. The pathogen replicates within an intracellular niche called an inclusion and is thought to rely heavily on host-derived proteins and lipids, including ceramide. Sortilin is a transmembrane receptor implicated in the trafficking of acid sphingomyelinase, which is responsible for catalysing the breakdown of sphingomyelin to ceramide. In this study, we examined the role of sortilin in *Chlamydia trachomatis* L2 development. Western immunoblotting and immunocytochemistry analysis revealed that endogenous sortilin is not only associated with the inclusion, but that protein levels increase in infected cells. RNAi-mediated depletion of sortilin, however, had no detectable impact on ceramide delivery to the inclusion or the production of infectious progeny. This study demonstrates that whilst *Chlamydia* redirects sortilin trafficking to the chlamydial inclusion, RNAi knockdown of sortilin expression is insufficient to determine if this pathway is requisite for the development of the pathogen.

## Abstract (precis)

**Summary:** The acid sphingomyelinase trafficking protein sortilin is modulated by chlamydial infection. RNAi-mediated depletion of sortilin does not, however, perturb chlamydial infection.

## INTRODUCTION

*Chlamydia trachomatis* is the most prevalent bacterial sexually transmitted infection amongst humans and the leading cause non-congenital blindness worldwide (Centers for Disease Control and Prevention, 2014; Belland et al., 2004). As an obligate intracellular pathogen, *C. trachomatis* passes its entire developmental cycle within an intracellular membrane-bound vacuole called the inclusion. The capacity to create and maintain such a specialised replicative niche is an important virulence property for many intracellular pathogens (Scott et al., 2002; Vergne et al., 2005; Dupont et al., 2009). To facilitate the growth of the inclusion and to ensure delivery of nutrients to the pathogen, *Chlamydia* manipulates the membrane trafficking pathways of the host cell (Elwell and Engel, 2012). Whilst there are indications that *Chlamydia* is capable of producing specific phospholipids autonomously (Yao et al., 2015), there is ample evidence to suggest that *Chlamydia* relies on host-derived lipids for survival such as sphingomyelin, phosphatidylcholine, phosphatidylinositol and cholesterol (Hatch and McClarty, 1998; Wylie et al., 1997; Hackstadt et al., 1995; Carabeo et al., 2003).

Using a short chain fluorescent analogue of ceramide (C_6_-NBD-ceramide), Hackstadt and co-workers demonstrated the conversion of ceramide to sphingomyelin in the Golgi and subsequent delivery and accumulation of the fluorescent lipid within the inclusion as early as 2 hours post-infection (hpi). (Hackstadt et al., 1996). Treatment with myriocin, a potent inhibitor of serine palmitoyltransferase, the initial enzyme in the biosynthesis of sphingomyelin (Hanada et al., 2000) perturbs homotypic fusion of chlamydial inclusions as well as inclusion membrane stability (Robertson et al., 2009). Concurrent treatment with dihydroceramide or sphingosine reversed this phenotype highlighting the requirement for ceramide and/or sphingomyelin in chlamydial biology (Robertson et al., 2009). *Chlamydia* are thought to acquire sphingomyelin by intercepting sphingomyelin-containing Golgi-derived exocytic vesicles destined for the plasma membrane (Hackstadt et al., 1996); fusion of multi-vesicular bodies with the inclusion (Beatty, 2008, 2006) or via non-vesicular means through the hijacking of the ceramide transfer protein (CERT) (Derre et al., 2011). With regards to the latter, Elwell and colleagues propose that *C. trachomatis* recruits CERT, its ER binding partner, VAP-A, and the sphingomyelin synthases, SMS1 and SMS2, to establish an ‘on-site sphingomyelin biosynthetic factory’ at or near the inclusion (Elwell et al., 2011). What is not clear is where the sphingomyelin precursor, ceramide, to support this system is sourced from.

The constitutive degradation of sphingolipids within the eukaryotic cell takes place in multi-vesicular bodies where sphingomyelin is converted to ceramide by acid sphingomyelinase (ASM) (Lansmann et al., 2003). ASM is demonstrated to play a central role in the cellular response to a variety of cellular stresses including environmental insults, apoptosis and pathogen infection (Smith and Schuchman, 2008). Sortilin is a transmembrane protein required for the trafficking of soluble ASM within the endosome/lysosome system (Willnow et al., 2008; Wahe et al., 2010). We speculated that sortilin-based trafficking of ASM may contribute to the supply of ceramide to the on-site sphingomyelin biosynthetic factory utilised by *Chlamydia*. We found that *C. trachomatis* L2 subverts the trafficking of sortilin to the inclusion leading to an increase in sortilin concentrations in infected cells. RNAi-mediated depletion of sortilin had no significant impact on chlamydial inclusion expansion or infectious progeny production.

## RESULTS

### Sortilin is upregulated in *C. trachomatis* L2 infected cells and is associated with the chlamydial inclusion

**Fig. 1.**
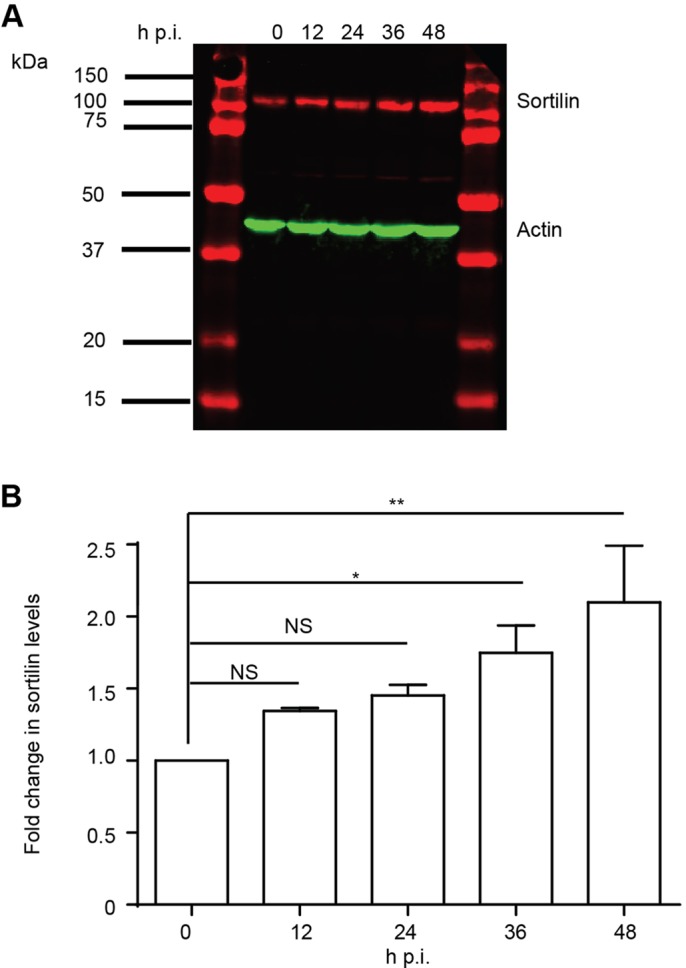
***Chlamydia* infection causes elevated levels of sortilin.** (A) Whole cell lysates of *C. trachomatis* L2-infected HeLa cells were harvested at indicated time points. 20 μg of proteins were subjected to western blot using rabbit polyclonal anti-sortilin and mouse monoclonal anti-beta actin antibodies followed by IR dye-conjugated secondary antibodies. Fluorescent intensities were detected by Li-Cor Odyssey Infrared Imaging System and (B) quantified using FIJI demonstrating a stark increase sortilin levels during infection. Error bars denote mean±s.e.m., *N*=2; one-way ANOVA; *P<0.05, **P<0.005.

**Fig. 2.**
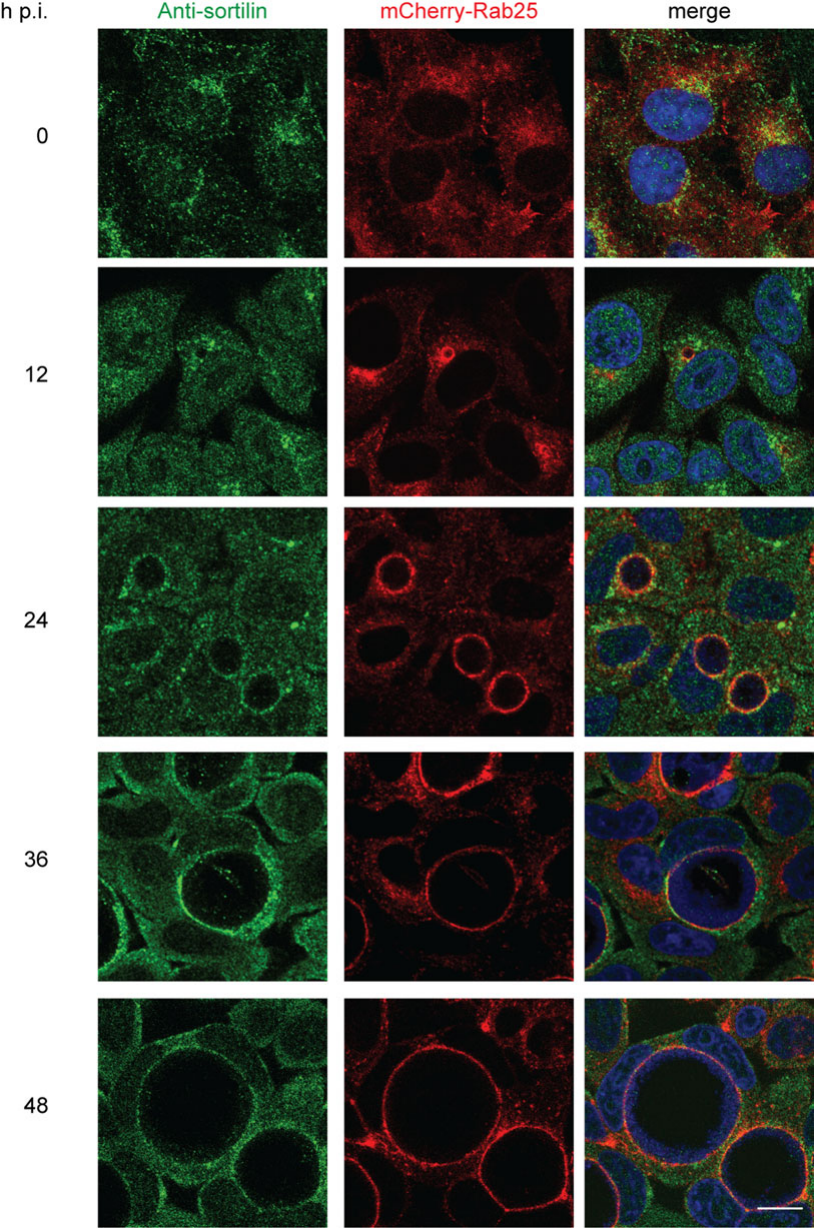
**Sortilin is associated with the inclusion throughout its development.** HeLa cells stably expressing mCherry-Rab25 were infected with CTL2 (MOI 0.5) for the indicated time points and fixed with 4% paraformaldehyde. Samples were then immunolabeled with anti-sortilin antibodies and appropriate secondary antibody and counter-stained with DAPI. Single optical sections were obtained using a Zeiss LSM710 meta confocal microscope with a 63× objective. Scale bar 20 µm.

To investigate a role for sortilin in *Chlamydia trachomatis* L2 (CTL2) infection, Western immunoblotting was initially performed to monitor the levels of sortilin in infected cells. Western immunoblotting of HeLa cells infected with CTL2 at a multiplicity of infection (MOI) ∼0.5 revealed an increase in total cellular sortilin levels from 12 hpi, with an approximately two-fold increase observed by 48 hpi relative to uninfected cells cultured under the same conditions (Fig. 1A,B). To determine if sortilin is associated with the outer membrane of the chlamydial inclusion throughout the course of infection, indirect immunofluorescence was performed using anti-sortilin antibodies to examine the subcellular distribution of sortilin in infected cells. An unpublished screen of Rab-fusion proteins (data not shown) revealed that mCherry-Rab25 localises with high fidelity to the CTL2 inclusion throughout the infection (Fig. S1). As such, we employed mCherry-Rab25 stably expressing HeLa cells to monitor the limiting membrane of the chlamydial inclusion at 12 h intervals. The samples were immunolabeled with anti-sortilin antibodies, followed by a fluorescently-conjugated secondary antibody, counter-stained with DAPI and examined using confocal microscopy (Fig. 2A). Consistent with previously published studies, in uninfected cells the majority of the endogenous sortilin-labelling was observed to localise to the perinuclear region of the cell, within the Golgi and in vesicles dispersed throughout the cytoplasm, (Kwon and Christian, 2011; Ni and Morales, 2006) presenting little overlap with mCherry-Rab25. During a chlamydial infection, however, endogenous sortilin is localised to the mCherry-Rab25-positive inclusions from 12 hpi. As the infection progressed, sortilin remained associated with the inclusion. Therefore, sortilin is efficiently transported to and remains associated with the chlamydial inclusion throughout the infection.

### Ceramide delivery to the inclusion is unaffected by depletion of sortilin

**Fig. 3.**
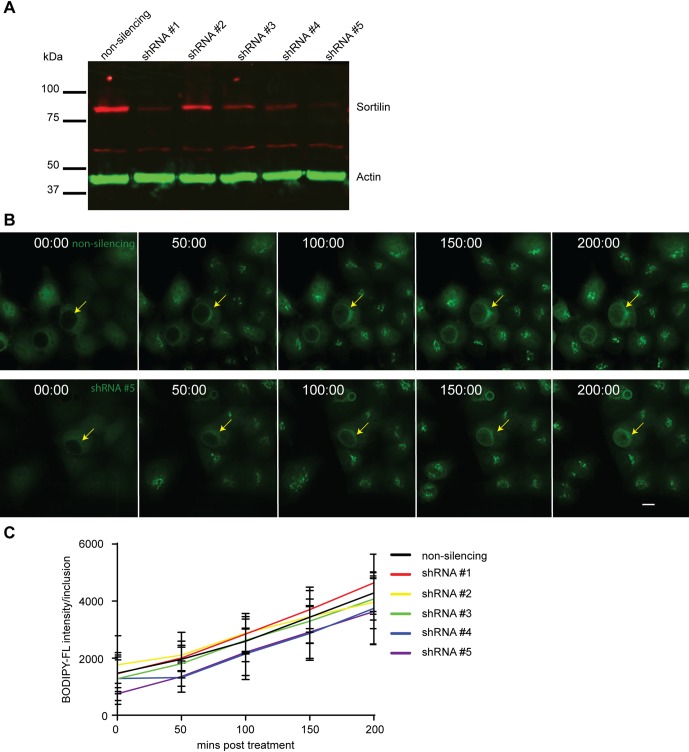
**Depletion of sortilin does not impact upon delivery of ceramide to the inclusion.** (A) The protein levels of sortilin in stable cell lines expressing non-silencing shRNA or shRNA targeting sortilin were analysed by western immunoblotting. (B) Sortilin knockdown cells were infected with CTL2 (MOI 0.5). At 23 hpi, cells were treated with BODIPY-FL-ceramide and delivery of ceramide followed for 4 h via live cell microscopy. Arrows highlight chlamydial inclusion. Images were captured using a Nikon Ti-E deconvolution microscope with a 40× objective. (C) Line graph represent the shift in mean fluorescent intensity per inclusion analysed by ImageJ. At least 9 inclusions were monitored. Error bar denote mean±s.e.m., *N*=2. Scale bar 20 µm.

To determine if delivery of sortilin to the chlamydial inclusion is required for infection progression, five shRNA-sortilin knockdown HeLa cell lines were generated. Western immunoblotting confirmed a >70% decrease in endogenous sortilin in four of these lines when compared with non-silencing control cells (Fig. 3A). BODIPY-FL-ceramide was used to monitor delivery of ceramide and its metabolites, which include sphingomyelin and glucosylceramide, in living cells using time-lapse videomicroscopy of sortilin knockdown cells infected with CTL2 (MOI ∼0.5) for 23 h. Initially the inclusion is void of any BODIPY-FL-ceramide or TurboGFP fluorescence (shRNA reporter), highlighting the barrier function of the inclusion membrane. By following the fluorescent ceramide analogue, we observe the rapid transportation of the lipid, first to the Golgi, and the subsequent accumulation of ceramide within the inclusion (Fig. 3B). Quantification of the accumulated fluorescent intensity within the inclusion over a period of 4 h revealed no detectable difference between the control and sortilin knockdown cells (Fig. 3C) confirming that delivery of BODIPY-FL-ceramide and its metabolites from the Golgi to the inclusion still occurs efficiently in these cells and that CTL2 is readily able to replicate.

### *Chlamydia* infection stabilises cellular sortilin concentrations

**Fig. 4.**
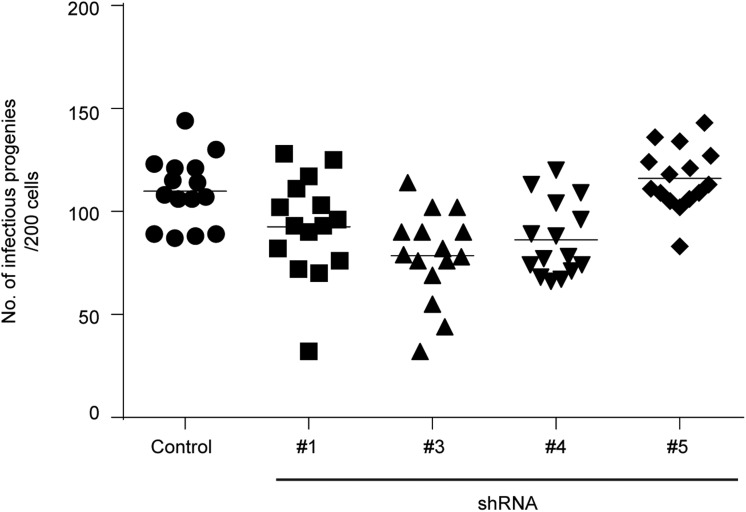
**Depletion of sortilin has no significant impact on the development of chlamydial infectious progeny.** Control and sortilin knockdown cells were seeded onto 96 well plates and infected with GFP-CTL2 for 48 h. Quantitative infectious progeny assays were performed using a Nikon Ti-E deconvolution microscope with a 20× objective.

Sortilin is required for the efficient trafficking of soluble cargo like ASM from the Golgi to endosomes and in cells depleted for sortilin the intracellular trafficking of ASM is impaired (Wahe et al., 2010; Ni and Morales, 2006). Delivery of ASM to the chlamydial inclusion would be required for the bacteria to gain access to ceramide generated from the conversion of sphingomyelin, a lipid transported to the inclusion from the Golgi (Hackstadt et al., 1995; Elwell et al., 2011). An infectious progeny assay was performed to assess the impact of sortilin knockdown upon the intracellular development of CTL2 using the four most significantly depleted sortilin knockdown cell lines. Briefly, control and sortilin knockdown cells were infected with GFP-expressing CTL2 for 48 h before lysing the cells. The lysates were then subjected to serial dilution and the diluents used to infect a new population of mCherry-Rab25-expressing HeLa cells for 24 h before being fixed, DAPI labelled and imaged using a Nikon Deconvolution microscope. We observed no significant impact in the growth of the primary inclusions or upon the development of infectious progeny (Fig. 4), implying that sortilin-dependent protein trafficking is not requisite for the intracellular development of *C. trachomatis* L2.

**Fig. 5.**
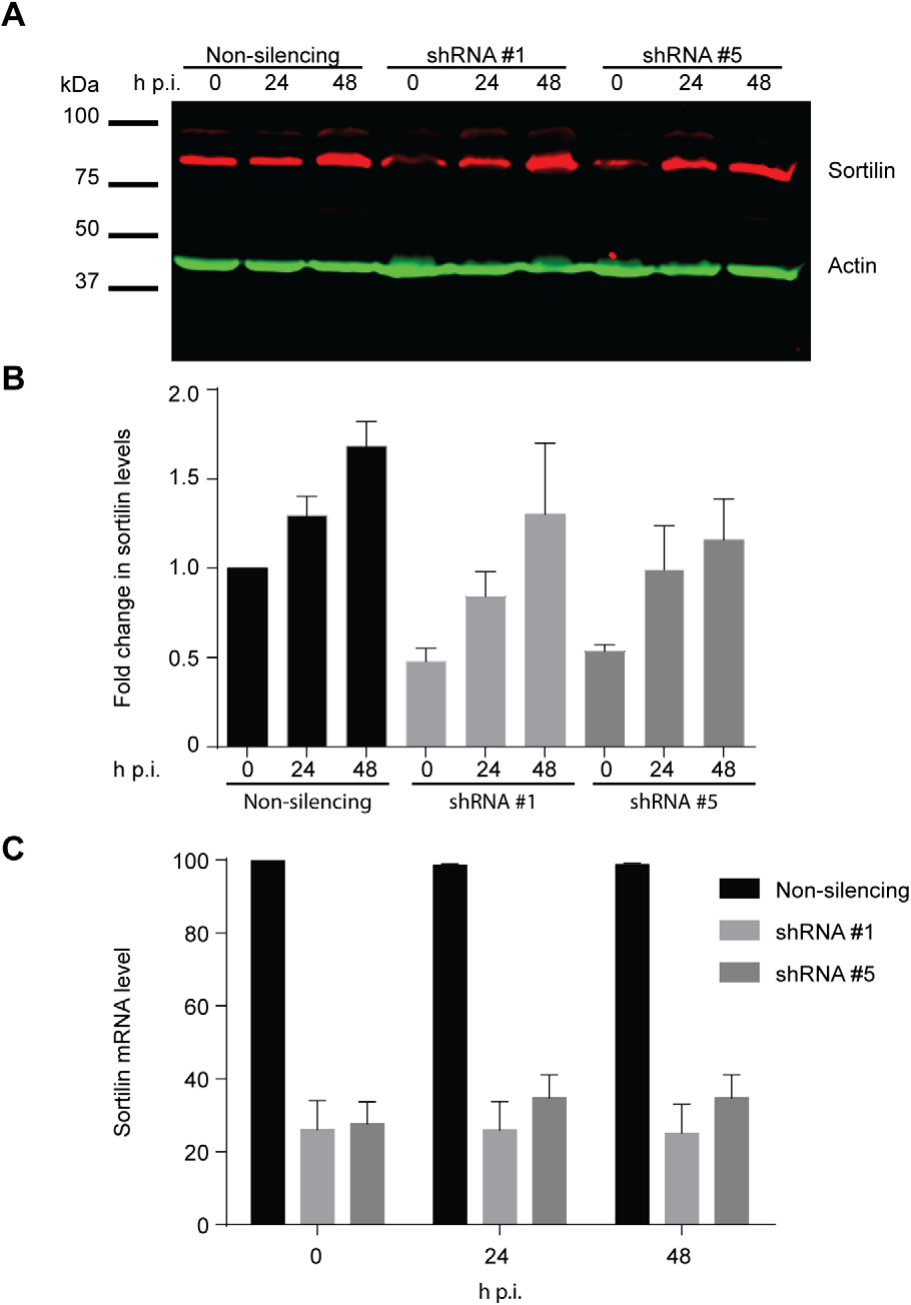
**The level of sortilin protein within infected knockdown cells increases to that observed within control cells.** (A) Non-silencing control and sortilin knockdown cells were infected and harvested at stipulated time points. 20 μg of proteins were subjected to western immunoblotting using rabbit polyclonal anti-sortilin and mouse monoclonal anti-beta actin antibodies followed by IR dye conjugated secondary antibodies. Fluorescent intensity captured by Li-Cor Odyssey Infrared Imaging System and (B) quantified using FIJI. (C) RNA from infected cells was extracted and prepared and quantified using Applied Science 7500 Real Time PCR Systems. Housekeeping gene GAPDH was used as an internal control. Error bars denote mean±s.e.m., *N*=2.

To further examine why RNAi-mediated depletion of sortilin had no impact on the intracellular growth and development of CTL2, we monitored the levels of sortilin in the two most significantly depleted knockdown lines throughout the course of infection. Surprisingly, at 24 hpi, sortilin levels in both knockdown lines had increased to levels comparable with control cells. This increase in sortilin levels was maintained throughout the infection (Fig. 5A,B). Analysis of sortilin mRNA levels by RT-PCR revealed no change during infection (Fig. 5C) suggesting that the observed increase in sortilin protein levels is a consequence of altered protein turnover rather than transcriptional regulation.

## DISCUSSION

Whilst living within a membrane-bound vacuole provides protection from the host cell's innate immune system it also presents inherent challenges, including the need to remodel the host's membrane trafficking pathways to generate a replicative niche that will expand and provide essential nutrients to support replication. The earliest studies of lipid acquisition by *Chlamydia* demonstrated that it is capable of acquiring lipids from the Golgi (Hackstadt et al., 1996, 1995). However, when infected cells were treated with Brefeldin A, an inhibitor of protein trafficking from the endoplasmic reticulum to the Golgi, only expansion of the inclusion membrane was impaired but not chlamydial growth or development (Hackstadt et al., 1996). This suggests that *Chlamydia* may be capable of acquiring host lipids required for replication from multiple sources.

Sphingomyelin is important for chlamydial growth and development. When infected cells are treated with myricon, a potent inhibitor of serine palmitoyltransferase, the initial enzyme in the biosynthesis of sphingomyelin, multiple small inclusions are observed suggesting host cell sphingomyelin is required for the homotypic fusion of inclusions (Robertson et al., 2009). Here we reveal that sortilin, which is responsible for transporting ASM to endosomes, is associated with the chlamydial inclusion (Fig. 1) and that sortilin levels increase dramatically through the course of a *C. trachomatis L2* infection. To dissect the role of sortilin in the context of a chlamydial infection, cells were depleted of endogenous sortilin using RNAi. When infected knockdown cells were treated with BODIPY-FL-ceramide, no discernible differences in its delivery to the inclusion compared to control cells were observed. Further, no impact on the development and production of infectious progeny was observed. These results suggest that the acquisition of ceramide, generated from sphingomyelin transported from the Golgi, by *Chlamydia* is independent of sortilin and the cargo proteins, including ASM, that it traffics. Indeed, ceramide acquisition by *Chlamydia* has been previously linked to CERT (Derre et al., 2011), Golgi fragmentation (Heuer et al., 2009) or interception of vesicles from the Golgi (Derre et al., 2011; Heuer et al., 2009; Hackstadt et al., 1996).

Further investigation revealed that when sortilin knockdown cells are infected with *C. trachomatis* L2, the protein levels of sortilin increased during the infection to levels similar to that of control cells. RT-PCR revealed no changes in sortilin mRNA levels, in either infected sortilin knockdown or control cells, indicating that sortilin transcriptional activity is not altered by the infection. Therefore, the increase in sortilin protein concentration observed in infected cells is likely a consequence of sequestration of sortilin within the inclusion. Sortilin sequestered within the chlamydial inclusion will not be exposed to degradative lysosomes preventing its normal turnover and newly synthesised sortilin will continue to be delivered to the inclusion from the Golgi. The resultant net increase in sortilin levels in spite of the introduced shRNAs indicates that RNAi-mediated depletion of sortilin is therefore unlikely to be an adequate tool for dissecting its possible role in chlamydial development. Normally sortilin is trafficked from the endosomal compartments to the Golgi via a retromer dependent retrograde trafficking pathway. While retromer was recently identified as enriched components on isolated *C. trachomatis* inclusions (Aeberhard et al., 2015), it remains unknown whether it forms functional transport carriers from the inclusion.

The possibility of functional redundancy in the trafficking of ASM also remains. Wahe et al. (2010) reported a significant reduction but not complete loss of prosaposin and ASM from phagosomes isolated from Sortilin^−/−^ macrophages, which is consistent with an alternate pathway for trafficking of these proteins to the phagosomes. The mannose-6 phosphate receptor also binds ASM and delivers it to endosomes (Ni and Morales, 2006) and has not only been associated with the chlamydial inclusion but has been proposed to be required for the attachment and internalisation of the pathogen (Vanooij et al., 1997; Puolakkainen et al., 2005). Its presence on the chlamydial inclusion may explain why depleting sortilin alone had no impact on the infection. Targeted disruption of sortilin and/or mannose-6 phosphate receptor may be worth investigating in the future.

## MATERIALS AND METHODS

### Constructs and reagents

Rabbit polyclonal antibodies against sortilin (Abcam, ab16640, 1:1000), rabbit monoclonal antibody against chlamydial protease/chaperone protein, CtHtrA (Huston et al., 2008) and mouse monoclonal antibodies against beta-actin (Merck Millipore, MAB1501, 1:1000) were used. Secondary antibodies were purchased from Molecular Probes (Life Technologies) and Li-Cor Bioscience. mCherry-Rab25 was obtained by performing restriction digest using restriction enzymes *Bam*HI and *Eco*RI on GFP-Rab25 (Casanova et al., 1999) to obtain the open reading frame of Rab25 and subcloned into mCherry-C1 following standard protocols. Stably expressing mCherry-Rab25 HeLa cells were generated by transient transfection of mCherry-Rab25 into HeLa cells using Lipofectamine 2000 as per manufacturer's instruction (Invitrogen). Transfected cells were selected using 400 µg/ml of G418 over a period of 14 days to generate stable cell lines expressing mCherry-Rab25. pGIPZ small hairpin RNA (shRNA) plasmids (Thermo Scientific) used included non-silencing control shRNA: RHS4346; human sortilin shRNAs (shRNA #1: V2LHS_31931, shRNA #2: V2LHS_31928, shRNA #3: V3LHS_359770, shRNA #4: V3LHS_359767, shRNA #5: V3LHS_359771) were supplied by the Institute for Molecular Bioscience Life Science Automation (LISA) Facility.

### Cell culture, transfection and generation of sortilin knockdown lines

HeLa wild-type (WT) and mCherry-Rab25 stably expressing cells were maintained in DMEM supplemented with 10% (v/v) FCS and 2 mM L-glutamine (Invitrogen) in a humidified air/atmosphere (5% CO_2_) at 37°C. Cell lines were routinely tested for mycoplasma contamination. Cells were seeded into 6 cm dishes and grown to 70% confluency prior to transfection. pGIPZ plasmids were transfected into cells using Lipofectamine 2000 as per manufacturer's instruction (Invitrogen). Transfected cells were selected for using 1 µg/ml puromycin over a period of 14 days to generate stable cell lines expressing individual shRNAs. shRNA-expressing cells were subsequently cultured in growth media containing 0.5 µg/ml puromycin.

### Quantitative RT-PCR

RNA from cells was extracted according to the manufacturer's instructions (Sigma-Aldrich). 1 µg of total RNA was used to produce cDNA using oligdT primers and Superscript III (Invitrogen). Quantitative RT-PCR was conducted using TaqMan gene expression assays for sortilin (SorCS1, Hs00364666_m1, Applied Biosciences) and the housekeeping gene glyceraldehyde 3-phosphate dehydrogenase (GAPDH, Hs99999905_m1, Applied Biosciences). GAPDH was used as an internal control to calculate the delta cycle threshold (ΔCT) for each sample.

### Chlamydial infection

*Chlamydia trachomatis* L2 (ATCC VR-902B) and GFP-expressing *Chlamydia trachomatis* L2 were generated as described previously (Wang et al., 2011). *Chlamydia* was used to infect cells at the indicated multiplicity of infection (MOI). Cells were infected for 2 h in normal DMEM growth media supplemented with 10% FCS (v/v) and 2 mM L-glutamine at 37°C humidified with 5% CO_2_. After which the media was replaced with fresh growth media and grown to the stipulated time points.

### Infectious progeny assay

Infectious progeny assay was performed as previously described (Gonzalez et al., 2014).

### Indirect immunofluorescence

HeLa cells grown on coverslips were transiently transfected with mammalian constructs, fixed and stained with indicated antibodies as described previously (Yang et al., 2015). The slides were imaged using a confocal laser scanning microscope (LSM 710, Zeiss) under 63× magnification. Data was processed using FIJI (http://fiji.sc/Fiji) and assembled using Adobe Illustrator CS5.

### Western immunoblotting

Cell monolayers were lysed directly with SDS lysis buffer (100 mM Tris/HCl, pH 6.8, 4% SDS, 20% glycerol, 0.02% bromophenol blue, 200 nM dithhiothreitol). Cell lysates were boiled at 95°C for 10 min. Equal amounts of protein were loaded and proteins resolved on 10% SDS-polyacrylamide gels and transferred onto Immobilon-FL PVDF membranes (Millipore, USA) according to the manufacturer's instructions. Western immunoblotting using ECL and Odyssey infrared imaging system (Li-Cor Biosciences) were performed as described previously (Yang et al., 2015). Fluorescence intensities were detected by Li-Cor Odyssey Infrared Imaging System (Li-Cor Biosciences). Protein analysis and quantification was performed using FIJI.

### BODIPY-FL-C5-ceramide labelling

Cells were seeded onto glass bottom 96 well dishes (MatTek Corporation) and infected with *C. trachomatis* L2 (MOI ∼0.5). At 23 hpi, the infected cells were treated with 1.25 µg/ml BODIPY-FL-C5-ceramide (Molecular Probes, B22650, 1:4000) as previously described (Pagano et al., 1991), and time-lapse videomicroscopy performed using an interval of 5 min on an inverted deconvolution microscope (Nikon Ti-E deconvolution, Nikon) under 40× magnification for 4 h.

### Statistical analysis

All data are presented as mean±s.e.m. and analysed with GraphPad Prism (GraphPad Software). For analysis of western blots, FIJI was used to quantify protein intensity and analysed with GraphPad Prism. To measure the delivery of BODIPY-FL-C5-ceramide to the chlamydial inclusion, nine inclusions were measured for each replicate. For infectious progeny assays, five images were captured on a Nikon Ti-E deconvolution microscope at 10× magnification and the number of inclusions quantified using FIJI. Final data represented the cumulative result from two independent experiments.
